# Supplementary material for: Pyruvate Dehydrogenase Kinase 1 inhibition mediated oxidative phosphorylation enhancement in cartilage promotes osteoarthritis progression
Source: BMC Musculoskelet Disord. 2023 Jul 20;24:597. doi: 10.1186/s12891-023-06585-6 (PMC10357736; doi:10.1186/s12891-023-06585-6)
Supplement: Supplementary file 1 — Additional file 1. [file 12891_2023_6585_MOESM1_ESM.zip › KEGG.Gsea.1653622432464/KEGG_PENTOSE_PHOSPHATE_PATHWAY.html]

Details for gene set KEGG\_PENTOSE\_PHOSPHATE\_PATHWAY[GSEA]

|  || Dataset | 55457\_collapsed\_to\_symbols.cls.cls #OA\_versus\_normal.cls.cls #OA\_versus\_normal\_repos |
| Phenotype | cls.cls#OA\_versus\_normal\_repos |
| Upregulated in class | OA |
| GeneSet | KEGG\_PENTOSE\_PHOSPHATE\_PATHWAY |
| Enrichment Score (ES) | 0.4260301 |
| Normalized Enrichment Score (NES) | 1.4555839 |
| Nominal p-value | 0.060185187 |
| FDR q-value | 0.087490566 |
| FWER p-Value | 0.897 |
Table: GSEA Results Summary

  

Fig 1: Enrichment plot: KEGG\_PENTOSE\_PHOSPHATE\_PATHWAY      
 Profile of the Running ES Score & Positions of GeneSet Members on the Rank Ordered List

  

| SYMBOL | TITLE | RANK IN GENE LIST | RANK METRIC SCORE | RUNNING ES | CORE ENRICHMENT || 1 | GPI | glucose-6-phosphate isomerase [Source:HGNC Symbol;Acc:HGNC:4458] | 166 | 0.842 | 0.0977 | Yes |
| 2 | RBKS | ribokinase [Source:HGNC Symbol;Acc:HGNC:30325] | 225 | 0.802 | 0.1986 | Yes |
| 3 | FBP1 | fructose-bisphosphatase 1 [Source:HGNC Symbol;Acc:HGNC:3606] | 772 | 0.576 | 0.2318 | Yes |
| 4 | ALDOC | "aldolase, fructose-bisphosphate C [Source:HGNC Symbol;Acc:HGNC:418]" | 870 | 0.551 | 0.2966 | Yes |
| 5 | DERA | deoxyribose-phosphate aldolase [Source:HGNC Symbol;Acc:HGNC:24269] | 975 | 0.523 | 0.3573 | Yes |
| 6 | PGLS | 6-phosphogluconolactonase [Source:HGNC Symbol;Acc:HGNC:8903] | 976 | 0.523 | 0.4260 | Yes |
| 7 | PFKM | "phosphofructokinase, muscle [Source:HGNC Symbol;Acc:HGNC:8877]" | 2260 | 0.331 | 0.3699 | No |
| 8 | TKTL1 | transketolase like 1 [Source:HGNC Symbol;Acc:HGNC:11835] | 2528 | 0.300 | 0.3885 | No |
| 9 | RPE | ribulose-5-phosphate-3-epimerase [Source:HGNC Symbol;Acc:HGNC:10293] | 3153 | 0.235 | 0.3709 | No |
| 10 | PRPS1 | phosphoribosyl pyrophosphate synthetase 1 [Source:HGNC Symbol;Acc:HGNC:9462] | 3717 | 0.184 | 0.3513 | No |
| 11 | FBP2 | fructose-bisphosphatase 2 [Source:HGNC Symbol;Acc:HGNC:3607] | 3763 | 0.180 | 0.3714 | No |
| 12 | PGM1 | phosphoglucomutase 1 [Source:HGNC Symbol;Acc:HGNC:8905] | 3990 | 0.157 | 0.3745 | No |
| 13 | ALDOA | "aldolase, fructose-bisphosphate A [Source:HGNC Symbol;Acc:HGNC:414]" | 4289 | 0.129 | 0.3682 | No |
| 14 | PRPS2 | phosphoribosyl pyrophosphate synthetase 2 [Source:HGNC Symbol;Acc:HGNC:9465] | 4365 | 0.123 | 0.3786 | No |
| 15 | TALDO1 | transaldolase 1 [Source:HGNC Symbol;Acc:HGNC:11559] | 5184 | 0.057 | 0.3227 | No |
| 16 | PFKP | "phosphofructokinase, platelet [Source:HGNC Symbol;Acc:HGNC:8878]" | 5500 | 0.032 | 0.3025 | No |
| 17 | PFKL | "phosphofructokinase, liver type [Source:HGNC Symbol;Acc:HGNC:8876]" | 5632 | 0.021 | 0.2951 | No |
| 18 | G6PD | glucose-6-phosphate dehydrogenase [Source:HGNC Symbol;Acc:HGNC:4057] | 7082 | -0.086 | 0.1939 | No |
| 19 | H6PD | hexose-6-phosphate dehydrogenase/glucose 1-dehydrogenase [Source:HGNC Symbol;Acc:HGNC:4795] | 8303 | -0.178 | 0.1226 | No |
| 20 | PRPS1L1 | phosphoribosyl pyrophosphate synthetase 1 like 1 [Source:HGNC Symbol;Acc:HGNC:9463] | 8382 | -0.184 | 0.1407 | No |
| 21 | ALDOB | "aldolase, fructose-bisphosphate B [Source:HGNC Symbol;Acc:HGNC:417]" | 8421 | -0.187 | 0.1623 | No |
| 22 | RPIA | ribose 5-phosphate isomerase A [Source:HGNC Symbol;Acc:HGNC:10297] | 9793 | -0.301 | 0.0954 | No |
| 23 | PGD | phosphogluconate dehydrogenase [Source:HGNC Symbol;Acc:HGNC:8891] | 10845 | -0.410 | 0.0676 | No |
| 24 | TKT | transketolase [Source:HGNC Symbol;Acc:HGNC:11834] | 12373 | -0.700 | 0.0409 | No |
Table: GSEA details [plain text format]

  

Fig 2: KEGG\_PENTOSE\_PHOSPHATE\_PATHWAY      
 Blue-Pink O' Gram in the Space of the Analyzed GeneSet

  

Fig 3: KEGG\_PENTOSE\_PHOSPHATE\_PATHWAY: Random ES distribution      
 Gene set null distribution of ES for **KEGG\_PENTOSE\_PHOSPHATE\_PATHWAY**
